# Interactions between *Parabacteroides goldsteinii* CCUG 48944 and diet ameliorate colitis in mice via regulating gut bile acid metabolism

**DOI:** 10.1002/imo2.70008

**Published:** 2025-03-18

**Authors:** Fujian Qin, Mengdi Zhang, Qingling Yang, Lei Wu, Tianxiao Mao, Xingchen Zhou, Jing Li, Maode Lai

**Affiliations:** ^1^ State Key Laboratory of Natural Medicines, School of Basic Medical Sciences and Clinical Pharmacy China Pharmaceutical University Nanjing China; ^2^ Department of Laboratory Medicine The First Affiliated Hospital of Nanjing Medical University Nanjing China; ^3^ Department of Pharmacy Zhongshan Hospital Fudan University Shanghai China; ^4^ Division of (Bio) Pharmaceutics Institute of Zhejiang University‐Quzhou Quzhou China; ^5^ School of Life Science and Technology China Pharmaceutical University Nanjing China; ^6^ Department of Pathology, Research Unit of Intelligence Classification of Tumor Pathology and Precision Therapy, Chinese Academy of Medical Science Key Laboratory of Disease Proteomics of Zhejiang University School of Medicine Hangzhou China

**Keywords:** bile acid, colorectal cancer, fiber free diet, inflammatory bowel disease, *Parabacteroides goldsteinii*

## Abstract

Inflammatory bowel disease (IBD) is a chronic disorder linked to an increased risk of colorectal cancer (CRC) and is characterized by significant dysbiosis in the gut microbiota. The commensal bacterium *Parabacteroides goldsteinii* (*P. goldsteinii*) has shown potential in modulating host metabolism and inflammatory responses. In this study, we investigated the probiotic properties of *P. goldsteinii* and its mechanism of action in IBD models, with a particular focus on bile acid metabolism and diet‐microbiota interactions. Fecal samples from patients with ulcerative colitis (*n* = 14), Crohn's disease (*n* = 22), and healthy controls (*n* = 13) were analyzed to assess *P. goldsteinii* relative abundance. In dextran sulfate sodium (DSS)‐induced colitis and azoxymethane (AOM)/DSS‐induced CRC mouse models, administration of *P. goldsteinii* significantly attenuated inflammation and tumorigenesis, particularly under fiber‐free diet conditions. Metabolomic profiling revealed an enrichment of secondary bile acids in *P. goldsteinii*‐treated mice, suggesting a link between bile acid metabolism and its anti‐inflammatory effects. Further mechanistic studies using bile salt hydrolase inhibitors and *Tgr5* knockout mice confirmed the role of bile acid regulation in mediating the therapeutic benefits of *P. goldsteinii*. Additionally, we found that dietary factors significantly influenced the colonization and metabolic activity of *P. goldsteinii*, thereby modulating its probiotic efficacy. This highlights the potential for microbiome‐based therapies tailored to specific dietary contexts in the treatment of IBD. Our findings demonstrate that *P. goldsteinii* can modulate gut bile acid metabolism to alleviate colitis, making it a promising candidate for probiotic applications in IBD management, with dietary modulation enhancing its therapeutic potential.

## Highlights

- The relative abundance of *Parabacteroides goldsteinii* (*P. goldsteinii*) significantly decreased in inflammatory bowel disease patients, and oral *P. goldsteinii* ameliorates dextran sulfate sodium (DSS)‐induced colitis and azoxymethane (AOM)/DSS‐induced colorectal cancer.
- *P. goldsteinii* treatment improves DSS‐induced intestinal metabolic disorders, especially bile acids.
- Diet determines the anti‐inflammatory effect of *P. goldsteinii*.

## Graphical abstract

In this study, we explored the therapeutic effects of *Parabacteroides goldsteinii* (*P. goldsteinii*) on colitis and its underlying mechanisms. Our results indicate a significant reduction of *P. goldsteinii* in the stools of inflammatory bowel disease patients. We demonstrate the therapeutic potential of *P. goldsteinii* in mitigating dextran sulfate sodium‐induced colitis and restraining tumorigenesis in the azoxymethane (AOM)/DSS mouse model under a fiber‐free diet. Metabolomic profiling further revealed an enrichment of fecal secondary bile acids in response to *P. goldsteinii*. By employing bile salt hydrolase inhibitors and Takeda G‐protein‐coupled receptor 5 (Tgr5) knockout mice, we established a connection between the anti‐inflammatory effect of *P. goldsteinii* and fecal bile acids. Furthermore, our findings highlight the modulatory role of diet in enhancing *P. goldsteinii*'s therapeutic potential, underlining the importance of diet in future probiotic research.

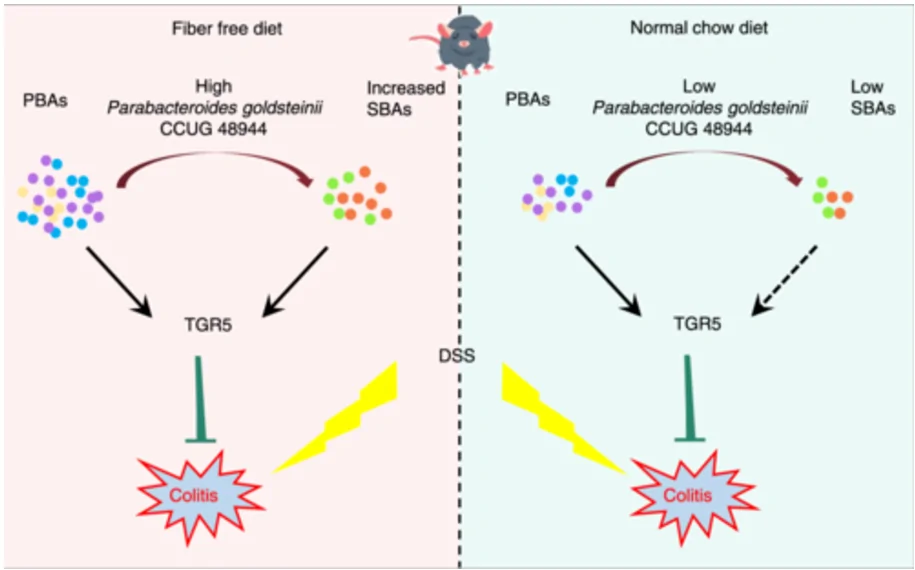

## 1 INTRODUCTION

Globally, inflammatory bowel disease (IBD), encompassing ulcerative colitis (UC) and Crohn's disease (CD), is becoming more prevalent. In the United States alone, IBD affects approximately 1.8 to 3.1 million individuals, resulting in significant healthcare costs [1]. In China, the number of IBD cases increased to 484,000 in males and 427,000 in females from 1990 to 2019, with projections indicating continued growth over the next 25 years [2]. Additionally, patients with longstanding or extensive IBD have an increased risk of developing colitis‐associated colorectal cancer (CRC), which has higher mortality rates than sporadic CRC [3, 4, 5]. Current IBD treatments primarily involve immune suppression, anti‐inflammatory, and biologics targeting cytokines (mainly TNF‐α) [6, 7]. However, many patients find these medications ineffective, ultimately requiring surgery [7, 8]. Thus, novel therapeutic strategies for IBD are urgently needed.

The pathophysiology of IBD is not fully understood, but accumulating evidence suggests that the commensal gut microbiota plays a crucial role. For instance, *Ruminococcus gnavus* [9, 10], *Fusobacterium nucleatum* [11] and *Enterococcus faecium* [12] promote colitis in mouse models, whereas *Lactobacillus plantarum* [13], *Bacteroides ovatus* [14], *Bifidobacterium adolescentis* [15] and *Bifidobacterium dentium* [16] attenuate intestinal inflammation via various mechanisms, including potent immunomodulatory properties and antioxidant effects.

Gut commensal *Parabacteroides goldsteinii* (*P. goldsteinii*) has been identified as a potential probiotic due to its antiobesity and anti‐inflammatory effects in mice [17]. Several antiobesity drugs, such as ethanol extract of propolis [18] and Isoliquiritigenin [19], have been proven to be associated with *P. goldsteinii*. Hirsutella sinensis polysaccharides have also been shown to improve high‐fat diet (HFD)‐induced obesity, inflammation, and insulin resistance by enriching *P. goldsteinii* [20]. Additionally, treatment with penicillin significantly increased the proportion of Treg cells in mice, potentially related to *P. goldsteinii* [21]. Recent research demonstrated that *P. goldsteinii* β‐hexosaminidase can induce differentiation of CD8aa‐expressing intraepithelial lymphocytes (CD4IELs) for anti‐inflammatory effects in mice [22]. Furthermore, oral administration of ophiocordyceps sinensis has been found to reduce colitis‐associated tumorigenesis, potentially due to the increased relative abundance of *P. goldsteinii* and *Bifidobacterium pseudolongum PV8‐2* [23]. Collectively, these findings suggest a protective role for *P. goldsteinii* in inflammatory conditions. Nevertheless, a direct causal link between *P. goldsteinii* and IBD pathogenesis has not yet been established.

Our research has discovered that the relative abundance of *P. goldsteinii* is markedly decreased in the feces of IBD patients. Importantly, we demonstrate that oral gavage of *P. goldsteinii* significantly reduced intestinal inflammation and neoplasia in mice. We further show that *P. goldsteinii* improves dextran sodium sulfate (DSS)‐induced fecal metabolic disorder, particularly bile acid metabolic profiles. Notably, we also found that diet dominates the anti‐inflammatory effect of *P. goldsteinii*. These findings highlight the importance of *P. goldsteinii* in protecting against colitis and tumorigenesis and provide insights into exactly understanding the convoluted interactions between colitis, CRC, and gut commensal microbiota.

## 2 RESULTS

### Reduced relative abundance of *Parabacteroides goldsteinii* in stool samples of IBD patients

**Figure 1:**
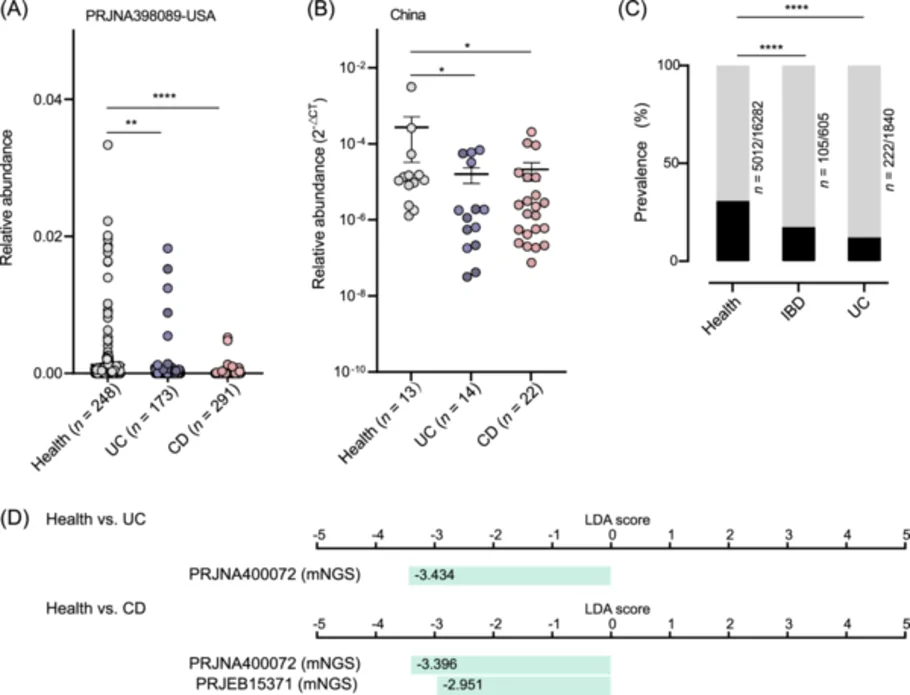
Parabacteroides goldsteinii (*P. goldsteinii*) decreases in stool samples of inflammatory bowel disease (IBD) patients. (A, B) Scatter diagrams illustrate the relative abundance of *P. goldsteinii* in stool samples from healthy controls, ulcerative colitis (UC), and Crohn's disease (CD) patients from two cohorts in the United States of America (USA) and China. Reference metagenomic sequences were obtained from public datasets (PRJNA398089). The relative abundance in Chinese samples was determined using quantitative real‐time polymerase chain reaction (qPCR). Data were presented as mean ± standard error of the mean (SEM), and statistical significance was assessed using the Mann–Whitney *U* test. (C) Prevalence of *P. goldsteinii* in the gut microbiome of IBD patients compared to healthy controls based on *GMrepo* data. (D) Marker‐centric view of *P. goldsteinii* across UC and CD; health‐enriched species are plotted in light green, using data from the *GMrepo* database. Differences in *P. goldsteinii* prevalence between healthy and diseased patients were assessed using the chi‐square test. Statistical significance: *p < 0.05, **p < 0.01, ***p < 0.001, ****p < 0.0001 compared to healthy control. mNGS, metagenomic next‐generation sequencing; LDA, Linear discriminant analysis.

Our initial analysis of published metagenomic cohorts (USA, PRJNA398089) revealed a significant decrease of *Parabacteroides goldsteinii* (*P. goldsteinii*) in patients with IBD (Figure 1A). To further investigate this association, we quantified *P. goldsteinii* in 49 stool samples from 14 UC patients, 22 CD patients, and 13 healthy controls using quantitative real‐time polymerase chain reaction (qPCR). The results confirmed a marked reduction of *P. goldsteinii* in IBD patients (Figure 1B). Consistent with our findings, analysis of data from *GMrepo* database [24] showed that *P. goldsteinii* is a marker taxon in the healthy group, with higher prevalence in healthy individuals (30.8%) than in IBD (17.4%) and colitis (12.1%) (Figure 1C,D). These results indicate a dysbiosis of fecal microbiota in IBD patients, characterized by a decrease in *P. goldsteinii*.

### Diet‐dependent alleviation of DSS‐induced colitis by oral *P. goldsteinii*

**Figure 2:**
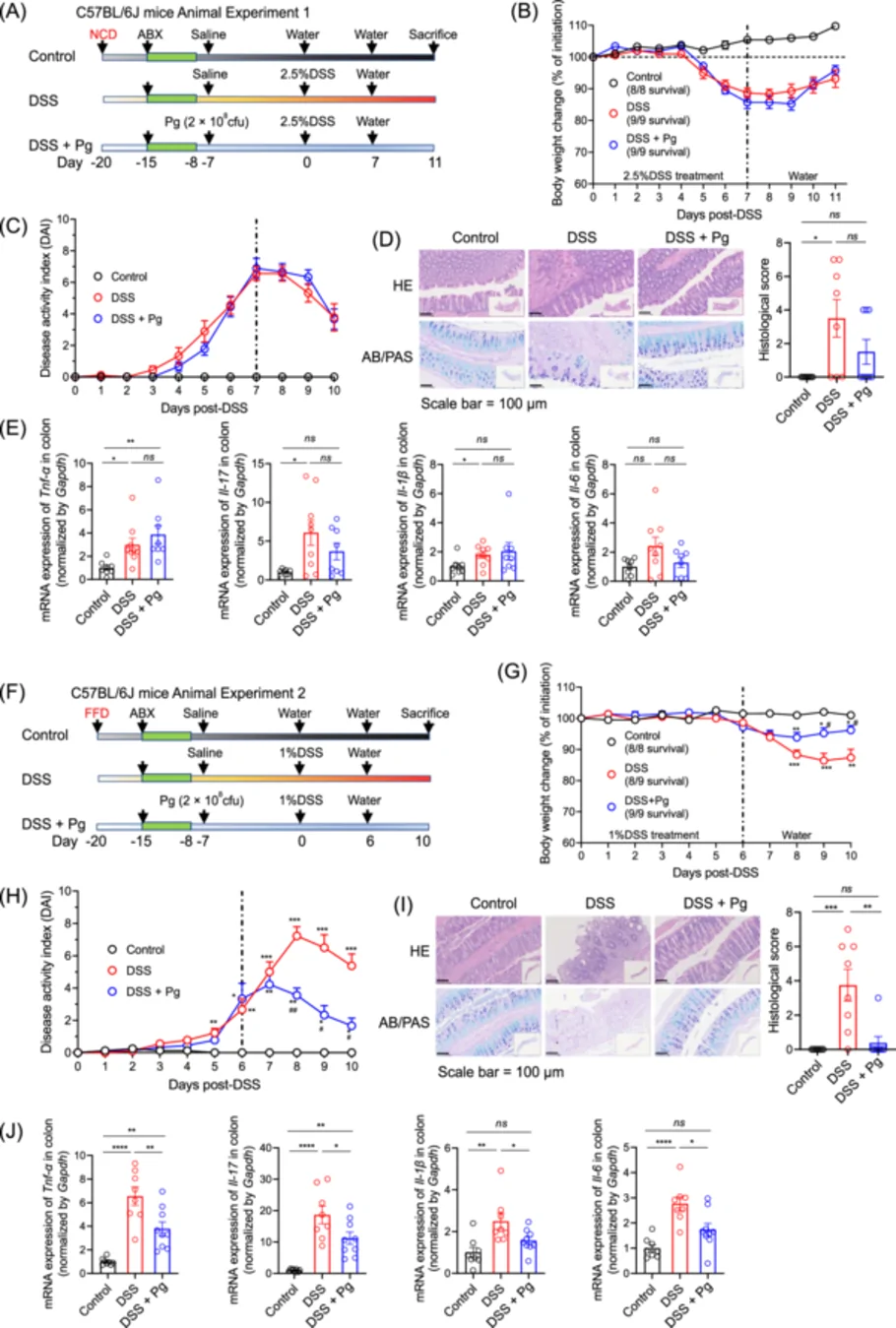
Oral gavage of *P. goldsteinii* (Pg) diet‐dependently alleviates dextran sulfate sodium (DSS)‐induced colitis. (A) Experimental design for the DSS‐induced colitis model on a normal chow diet (NCD). (B) Body weight changes after DSS administration. (C) Disease activity index (DAI) scores post‐DSS drinking. (D) Images of the representative HE and Alcian blue/periodic‐acid Schiff (AB/PAS) staining of the distal colon and histology score are shown (scale bar, 100 μm). HE: *n* = 8 mice/group; AB/PAS: *n* = 5 mice/group. (E) Relative mRNA levels of pro‐inflammatory cytokine‐related genes in the colon. *n* = 8−9 mice/group. (F) Experimental design for the DSS‐induced colitis model on a fiber‐free diet (FFD). (G) Body weight changes post‐DSS drinking. (H) DAI score after DSS administration. Data were analyzed in (G) and (H) using two‐way analysis of variance (ANOVA) (Tukey's multiple comparision test), with group factor and time as factors. *p < 0.05, **p < 0.01, ***p < 0.001 (DSS group or DSS + Pg group vs. Control group); ^#^ *p* < 0.05, ^##^ *p* < 0.01 (DSS group vs. DSS + Pg group). (I) Representative images of HE and AB/PAS staining of the distal colon and histology score are shown (scale bar, 100 μm). HE: *n* = 8 mice/group; AB/PAS: *n* = 5 mice/group. (J) Colonic mRNA expression of pro‐inflammatory‐related genes. *n* = 8−9 mice/group. Statistical significance in (D), (E), (I), and (J) were determined by one‐way ANOVA with Tukey's multiple comparision test. *p < 0.05, **p < 0.01, ***p < 0.001, ****p < 0.0001, *ns* indicates no significance. *Gapdh*, glyceraldehyde‐3‐phosphate dehydrogenase.

To investigate the possible impact of *P. goldsteinii* on the progression of colitis in mice fed a normal chow diet (NCD), an antibiotics cocktail (ABX) was administered to eliminate gut bacteria, as previously reported [25, 26] (Figure 2A). Mice treated with *P. goldsteinii* showed no significant improvement in colitis, as indicated by similar body weights (Figure 2B), disease activity index (DAI) scores (Figure 2C), and histology score (Figure 2D) compared to the DSS group. There were no notable changes in pro‐inflammatory cytokine expression in the colon (Figure 2E). Protein levels of ZO‐1 and Occludin in the colon also remained unchanged (Figure S1).

Given previous studies have revealed that a high‐fiber diet reinforces the pro‐inflammatory effects of *Segatella copri* (equivalent *Prevotella copri*) [27], we examined the effects of *P. goldsteinii* on colitis in mice fed a fiber‐free diet (FFD) (Figure 2F). Mice treated with *P. goldsteinii* exhibited less weight loss compared to the DSS group (Figure 2G). The DAI was also significantly lower in *P. goldsteinii*‐gavaged mice than in saline‐fed mice (Figure 2H). At harvest, the colon in the DSS group exhibited more severe inflammatory infiltration, marked crypt loss, epithelial layer destruction, and ulcerative lesions. In contrast, mucosal inflammation was significantly reduced in *P. goldsteinii*‐colonized mice (Figure 2I). Additionally, goblet cell numbers were restored (Figure 2I), and pro‐inflammatory cytokine (*Tnf‐α*, *Il‐1β*, *Il‐6*, and *Il‐17*) expression was decreased in colon tissues (Figure 2J). Immunohistochemical analysis showed increased levels of ZO‐1 and Occludin, indicating enhanced intestinal barrier function after *P. goldsteinii* intervention (Figure S2). These results demonstrated that colonization of pseudo‐sterile mice with *P. goldsteinii* is sufficient to improve DSS‐induced colitis under FFD conditions.

Diet, especially fiber, has been proven to alter gut microbiota [28, 29]. So next, we assessed *P. goldsteinii* colonization efficiency under different diets by measuring its relative abundance in fecal samples over time (Figure S3A). FFD‐fed mice treated with *P. goldsteinii* maintained normal body weights (Figure S3B) and showed increased *P. goldsteinii* relative abundance in feces (Figure S3C). Moreover, *P. goldsteinii*‐specific probes (Figure S3D and Table S1) were designed, and fluorescence in situ hybridization confirmed higher colonization in the colonic epithelium of FFD‐fed mice (Figure S3E). In summary, these findings suggest that the anti‐inflammatory effect of *P. goldsteinii* is diet‐dependent, influenced by differences in colonization efficiency.

### Inhibition of colorectal tumorigenesis by *P. goldsteinii* in azoxymethane (AOM)/DSS mouse models

**Figure 3:**
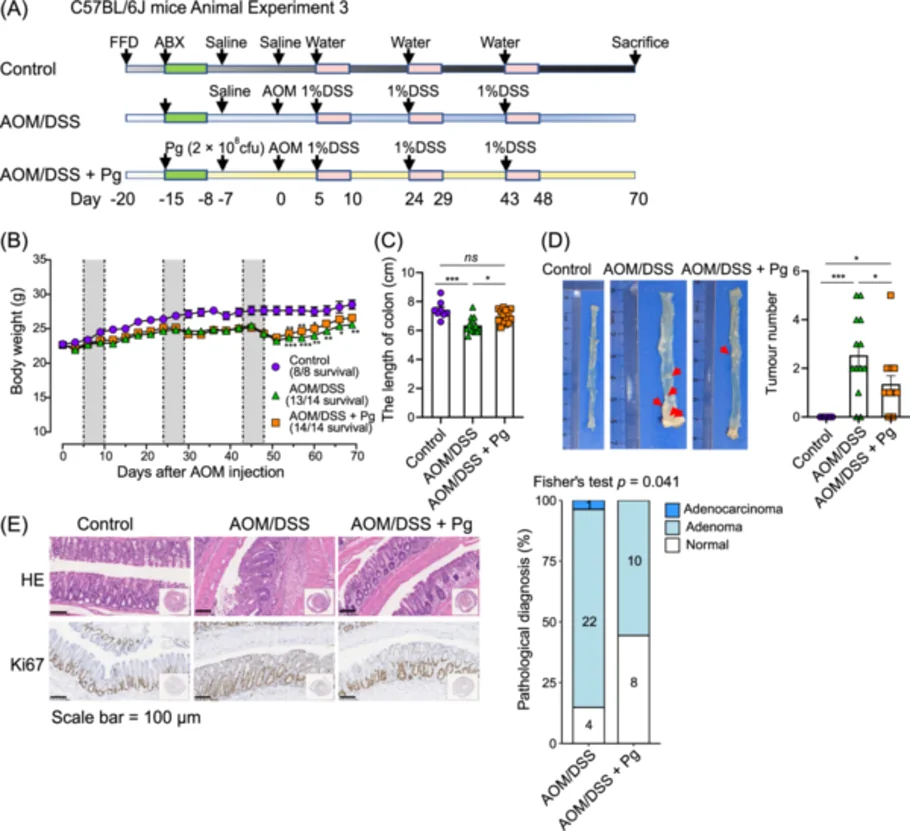
*P. goldsteinii* suppress azoxymethane (AOM)/dextran sulfate sodium (DSS)‐induced colorectal tumorigenesis in mice. (A) Experimental scheme of AOM/DSS‐induced CRC model. (B) Body weight changes after AOM injection. *p < 0.05, **p < 0.01, ***p < 0.001 (AOM/DSS group or AOM/DSS + Pg group vs. Control group, two‐way ANOVA with Tukey's multiple comparision test). (C) Colon length measurements. (D) Representative colon images and tumor counts, with red arrows indicating adenomas. Significance in (C) and (D) were determined by one‐way ANOVA with Tukey's multiple comparision test. *p < 0.05, ***p < 0.001, *ns* indicates no significance. (E) Representative images of HE and Ki67 staining of the large intestine, with histology scores (scale bar, 100 μm). HE: *n* = 8−14 mice/group; Ki67: *n* = 5−7 mice/group. Differences in tumor differentiation between AOM/DSS group and AOM/DSS + Pg group were assessed using the Fisher's test. CRC, colorectal cancer.

IBD may lead to diminished quality of life and reduced life expectancy, accompanied by heightened susceptibility to CRC [5]. Given the observed anti‐inflammatory effects of *P. goldsteinii* in the DSS‐induced colitis model, we explored its potential to prevent colitis‐associated tumorigenesis in mice (Figure 3A). Mice treated with *P. goldsteinii* had higher body weights (Figure 3B), longer colon lengths (Figure 3C), and fewer tumors (Figure 3D) compared to the AOM/DSS group. The presence of colonic adenomas and adenocarcinomas was confirmed by a pathologist through microscopic histological examination. Histological analysis confirmed a lower histology score and fewer Ki67‐positive cells in *P. goldsteinii*‐treated mice, suggesting its tumor‐suppressive role in AOM/DSS models (Figure 3E).

### *P. goldsteinii* improved fecal metabolic disorders induced by DSS

**Figure 4:**
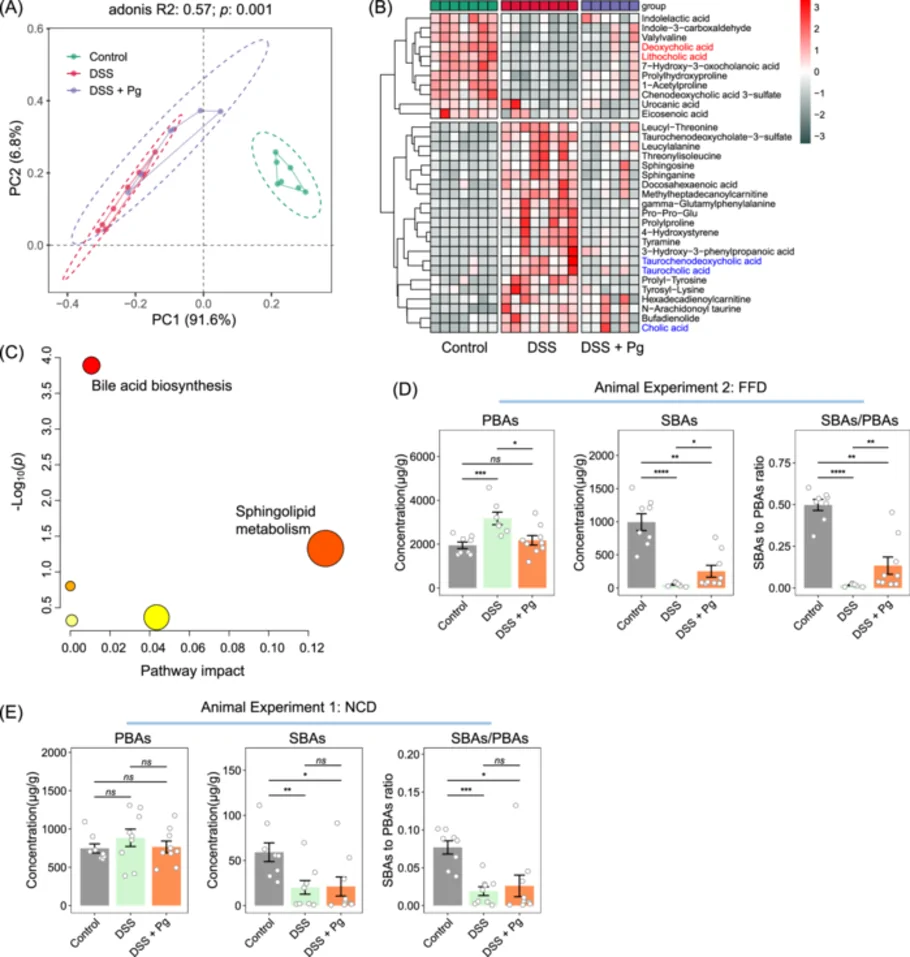
*P. goldsteinii* improves fecal metabolism in dextran sulfate sodium (DSS)‐treated mice. (A) Principal component analysis (PCA) plot of alterations in fecal metabolites among three groups of mice (Animal Experiment 2, permutational multivariate analysis of variance (PERMANOVA) test). *n* = 6−8 mice/group. (B) Heatmap shows the relative abundance of key differential metabolites among the control, DSS, and DSS + Pg groups. (C) Pathway enrichment analysis using MetoboAnalysis 5.0. (D) Absolute concentration of fecal primary bile acids (PBAs), secondary bile acids (SBAs), and SBAs/PBAs ratio in mice on a fiber‐free diet (FFD, Animal Experiment 2). *n* = 8−9 mice/group. (E) Absolute concentration of fecal PBAs, SBAs, and the ratio of SBAs/PBAs in mice on a normal chow diet (NCD, Animal Experiment 1). *n* = 8−9 mice/group. Statistical significance in (D) and (E) were determined by one‐way ANOVA with Tukey's multiple comparision test. *p < 0.05, **p < 0.01, ***p < 0.001, ****p < 0.0001, *ns* indicates no significance.

To gain a preliminary understanding of the mechanism in which *P. goldsteinii* ameliorates intestinal inflammation, we next analyzed fecal metabolites in IBD mice using ultra‐performance liquid chromatography‐quadrupole time‐of‐flight mass spectrometry (UPLC‐Q/TOF‐MS) (Animal Experiment 2) (Figure S4). PCA analysis showed that *P. goldsteinii* partially restored the metabolite profile of DSS‐treated mice toward that of control mice (Figure 4A). Subsequently, the orthogonal projection to latent structure discriminant analysis shows an obvious distinction between the control and DSS groups. Select differential metabolites based on the false discovery rate < 0.05, the absolute value of log_2_ fold‐change > 1, and variable importance in the projection > 1. (Figure S5). We further identified 33 metabolites of *P. goldsteinii* pullback by secondary ion comparison to the Human Metabolome Database (Figure 4B). The altered metabolites were mainly enriched in the phospholipid metabolism and bile acid synthesis pathways, which were analyzed by the MetaboAnalyst database (https://www.metaboanalyst.ca/) (Figure 4C). Intriguingly, bile acids have been reported to play an important role in the progression of IBD, which are important regulators of lipid metabolism. Therefore, the alterations of bile acid in stool were further confirmed by targeted mass spectrometry. Of note, primary bile acids (PBAs) were substantially downregulated (taurocholic acid (TCA), glycocholic acid (GCA), cholic acid (CA), taurochenodeoxycholic acid (TCDCA), tauro β muricholic acid, tauroursodeoxycholic acid (TUDCA), ursodeoxycholic acid (UDCA)) and secondary bile acids (SBAs) (deoxycholic acid (DCA), lithocholic acid (LCA), ω muricholic acid (ωMCA)) enriched in *P. goldsteinii*‐treated groups compared to the DSS group under FFD (Figure 4D, Figure S6). However, no significant changes were observed under NCD conditions (Figure 4E, Figure S7), indicating a diet‐dependent effect on bile acid metabolism. Altogether, the above results suggest that *P. goldsteinii* colonization recovers fecal metabolism in mice, especially bile acids, which might contribute to its improvement in colitis.

### Bile salt hydrolase (BSH) inhibitor eliminated the anti‐inflammatory effect of *P. goldsteinii*

**Figure 5:**
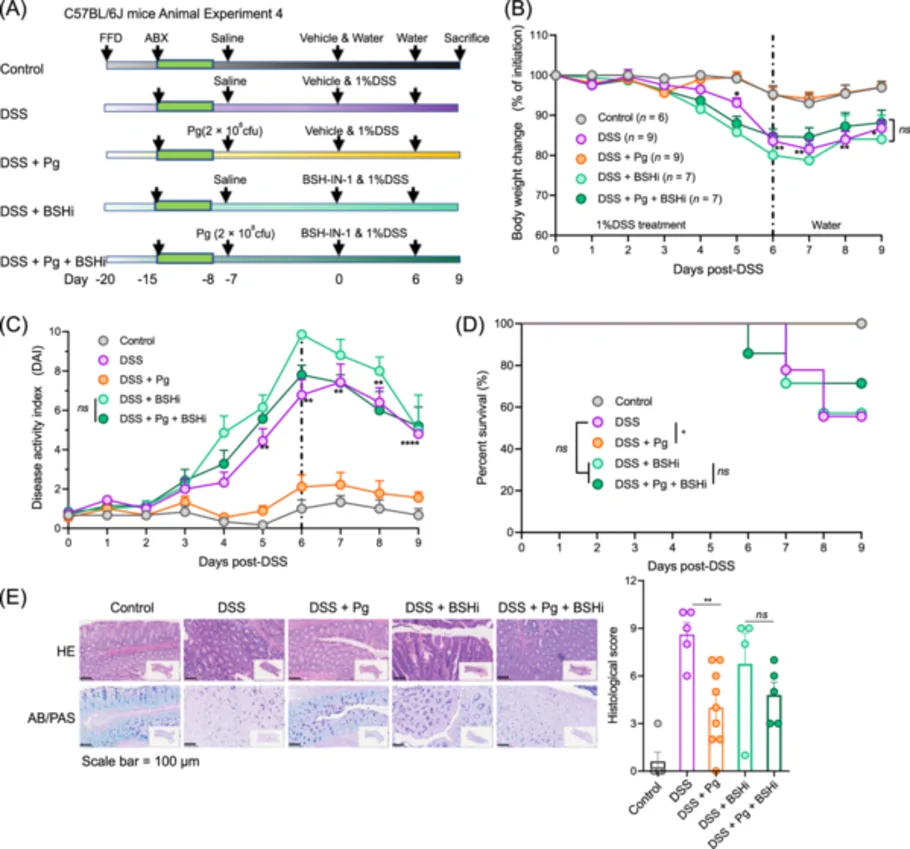
Anti‐inflammation function of *P. goldsteinii* is abrogated by bile salt hydrolase inhibitor (BSHi). (A) Experimental design for dextran sulfate sodium (DSS)‐induced colitis model with *P. goldsteinii* and/or BSH‐IN‐1 treatment. (B) Body weight changes post‐DSS drinking. (C) Disease activity index (DAI) score post‐DSS drinking. Data were analyzed in (B) and (C) using two‐way ANOVA with Tukey's multiple comparision test. *p < 0.05, **p < 0.01, ****p < 0.0001 (DSS group vs. DSS + Pg group); *ns* indicates no significance (DSS + BSHi group vs. DSS + BSHi + Pg group). (D) Survival rates in mice. *p < 0.05, *ns* indicates no significance (log‐rank test). (E) Representative images of HE and AB/PAS staining of the distal colon and histology score are shown (scale bar, 100 μm). HE: *n* = 4−9 mice/group; AB/PAS: *n* = 4−5 mice/group.

BSH is a key metabolic enzyme that catalyzes the conversion of conjugated PBAs to SBAs, and our previous results indicated that BSH is also found in *Parabacteroides* [30]. We investigated the effect of bile acids on the anti‐inflammatory activity of *P. goldsteinii* using a BSH inhibitor (BSH‐IN‐1) (Figure 5A). The inhibitor negated the weight improvement (Figure 5B), DAI score reduction (Figure 5C), and increased survival (Figure 5D) observed with *P. goldsteinii* treatment. Histological analysis confirmed reduced protection in BSH‐IN‐1‐treated mice (Figure 5E), suggesting that *P. goldsteinii*'s benefits in colitis remission are BSH‐dependent.

### *P. goldsteinii* ameliorated DSS‐induced colitis depends on Takeda G‐protein‐coupled receptor 5 (TGR5)

**Figure 6:**
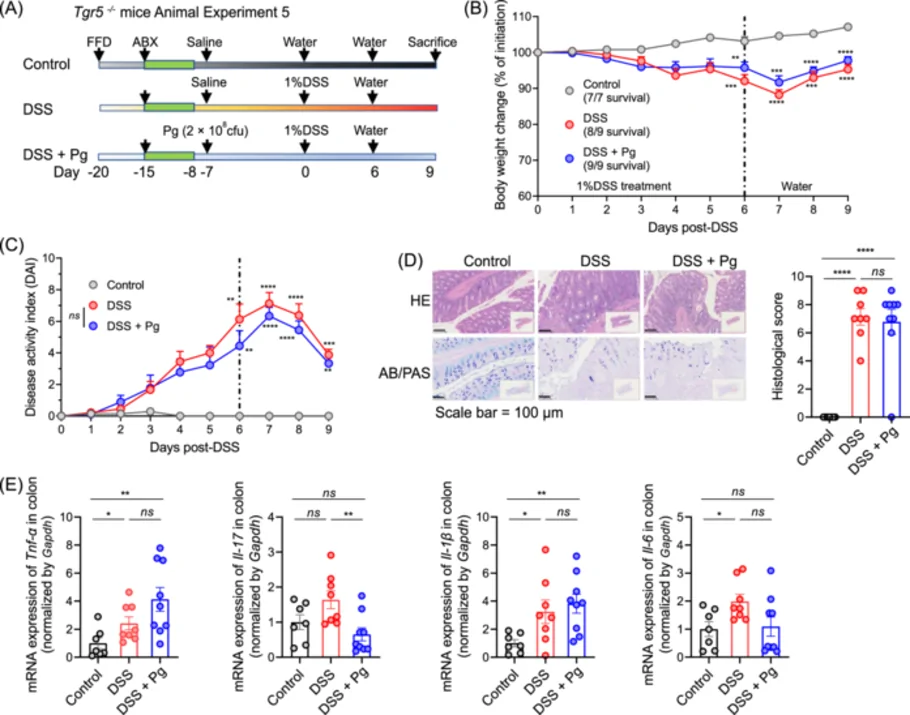
Protective effect of *P. goldsteinii* on dextran sulfate sodium (DSS)‐induced colitis is lost in *Tgr5* deficient mice. (A) Experimental design for DSS‐induced colitis model in *Tgr5* ^−/−^ mice (on a C57BL/6J background). (B) Body weight changes post‐DSS drinking. (C) Disease activity index (DAI) score post‐DSS drinking. Data were analyzed in (B) and (C) using two‐way ANOVA with Tukey's multiple comparision test. **p < 0.01, ***p < 0.001, ****p < 0.0001 (DSS group or DSS + Pg group vs. Control group); *ns* indicates no significance. (D) Images of the representative HE and AB/PAS staining of the distal colon and histology score are shown (scale bar, 100 μm). HE: *n* = 7−9 mice/group; AB/PAS: *n* = 5 mice/group. (E) mRNA expression levels of pro‐inflammatory cytokines in the colon. Statistical significance in (D) and (E) were determined by one‐way ANOVA with Tukey's multiple comparision test. *p < 0.05, **p < 0.01, ***p < 0.001, ****p < 0.0001, *ns* indicates no significance.

To further elucidate whether *P. goldsteinii* improves enteritis by regulating bile acids, the mRNA levels of bile acid receptors in the colon, including Farnesoid X receptor (*Fxr*), Takeda G‐protein‐coupled receptor 5 (*Tgr5*), Pregnane X receptor (*Pxr*), Vitamin D receptor (*Vdr*), and Constitutive androstane receptor (*Car*), were detected. We observed that among these receptors, *Tgr5* mRNA was significantly elevated after *P. goldsteinii* treatment (Figure S8). Given TGR5's role in weight loss and epithelial regeneration [31, 32, 33, 34], we tested the effects of *P. goldsteinii* in *Tgr5*‐deficient mice (on a C57BL/6J background, Figure 6A). Genotypes were identified by PCR using tail DNA (Figure S9). Noteworthy, the improvements in body weight and DAI by *P. goldsteinii* were blunted when *Tgr5* was knocked out (Figure 6B,C). Additionally, colon histological results showed that the therapeutic effects of *P. goldsteinii* were impaired in the *Tgr5* knock‐out mice, similar to the DSS group (Figure 6D). Consistently, the alleviating effects of main pro‐inflammatory genes (*Tnf‐α*, *Il‐1β*, and *Il‐6*) levels in the *P. goldsteinii* group were also blunted in *Tgr5* deficient mice (Figure 6E), confirming the critical role of bile acids in *P. goldsteinii's* therapeutic effects on IBD. Interestingly, *P. goldsteinii* intervention was still effective in inhibiting *Il‐17* expression (Figure 6E). In conclusion, the TGR5 signaling is essential for the IBD‐suppressive effect of *P. goldsteinii*.

## 3 DISCUSSION

In this study, we demonstrated a significant reduction of *Parabacteroides goldsteinii* (*P. goldsteinii*) in patients with IBD, both in previously published cohorts and in samples from the First Affiliated Hospital of Nanjing Medical University (Figure 1). The gut commensal microbiota is tightly linked to the pathogenesis of IBD. Consequently, we performed experiments involving the protection of *P. goldsteinii* on IBD using DSS‐induced mouse mode (Figure 2). Our studies reveal that *P. goldsteinii* dramatically inhibited the development of DSS‐induced colitis in FFD‐fed mice. Furthermore, oral treatment of AOM/DSS‐induced CRC mice with live *P. goldsteinii* abolished colorectal tumourigenesis (Figure 3), hence confirming its protective role against IBD and CRC in mice. Consistently, previous studies also reported that *P. goldsteinii* has a beneficial effect on anastomotic leak in CRC surgery due to its anti‐inflammatory properties [35]. Moreover, coculture experiments demonstrated that *P. goldsteinii* directly inhibited HCT116 and SW620 cell growth [36]. These results collectively demonstrated that *P. goldsteinii* is a potential probiotic for combating IBD and CRC.

A key finding of this study is the link between the anti‐inflammatory effects of *P. goldsteinii* and bile acid metabolism. Bile acids, which are categorized into PBAs and SBAs, are modulated by gut microbiota [37]. Imbalances in these bile acid pools have been associated with colitis, with PBAs such as CA and CDCA enriched in IBD patients, while SBAs such as DCA and LCA are depleted [38, 39]. Similarly, we observed the same trend in the DSS‐induced colitis model, and *P. goldsteinii* partially reverted bile acid dysfunction (Figure 4). Recently, studies have shown that CA can promote colitis by suppressing Lgr5^+^ intestinal stem cell regeneration [40], while LCA and DCA ameliorate intestinal inflammation, possibly through TGR5 [41]. Secondary bile acids produced by *Parabacteroides distasonis* have also been linked to weight loss in HFD‐fed mice [42]. Our use of BSH inhibitors and *Tgr5* knockout mice further demonstrated that *P. goldsteinii*‐mediated anti‐inflammatory effects are dependent on its ability to modulate bile acid metabolism (Figures 5, 6). These findings highlight the potential of *P. goldsteinii* to boost secondary bile acid production, suggesting that this strain may serve as a probiotic by leveraging bile acid pathways to reduce inflammation.

Remarkably, we discovered that diet affects the efficacy of *P. goldsteinii*. A previous study reported that experimental diets dictate *Lactobacillus*' metabolic benefits [43]. A clinical trial demonstrates a response to the probiotic supplement (*Limosilactobacillus reuteri NCIMB 30242, Lactiplantibacillus plantarum UALp‐05™*, and *Bifidobacterium animalis subsp. lactis B420™*) intervention for metabolic syndrome that may correspond to diet [44]. Indeed, the gut microbiota is regulated by a combination of diet, host genetics, and gender influences [36, 45, 46, 47]. For instance, *Roseburia intestinalis*, a butyrate‐producing bacterium, is highly responsive to the host diet [48], and *Parabacteroides* has been identified as a diet‐specific keystone organism [46]. Our findings showed that *P. goldsteinii* was more highly colonized in FFD‐fed mice (Figure S3), which had higher levels of SBAs in their feces (Figure 4). In this context, we found that FFD‐fed mice are indeed more sensitive to DSS (Figure 2), consistent with previous clinical results [49]. This may be due to altered microbial composition and changes in intestinal motility, as reported in earlier studies [28]. Overall, the discrepancy in the effect of *P. goldsteinii* on different diets may be in part explained by colonization efficiency and metabolism.

Despite the promising results, our study has several limitations. We used the type strain *P. goldsteinii* CCUG 48944, and it would be beneficial to evaluate whether similar effects can be observed with clinically isolated strains. Still, our findings suggest that bacterial colonization and metabolism may influence the anti‐inflammatory activity of *P. goldsteinii* in the presence of cellulose, but further validation is required. Finally, research should also explore the impact of other prebiotics on *P. goldsteinii* and the mechanisms by which *P. goldsteinii* affects CRC to facilitate its clinical application.

## 4 CONCLUSION

In conclusion, our study reveals a significant reduction of *P. goldsteinii* in IBD patients and demonstrates that its administration can mitigate DSS‐induced colitis by regulating gut bile acid metabolism. Notably, dietary fiber was found to impair the anti‐inflammatory effect of *P. goldsteinii*, suggesting that its use as a probiotic supplement for IBD treatment should be accompanied by rational dietary interventions. Future preclinical and clinical studies should fully consider the impact of diet on probiotic efficacy to maximize therapeutic outcomes.

## 5 METHODS

### Quantitation of *P. goldsteinii* relative abundance in feces

To detect the relative abundance of *P. goldsteinii*, bacterial DNA was extracted from human and mouse feces using QIAamp Fast DNA Stool Kit (Cat# 51604; QIAGEN) and Solarbio Stool Genomic DNA Extraction Kit (Cat# D2700; Solarbio), respectively, following the manufacturer's instruction. Quantification was performed on a QuantStudio3 qPCR machine using ChamQ SYBR qPCR Master Mix (Cat# Q711‐02; Vazyme Biotech). The 2^(−ΔCt) method was employed for relative abundance calculation, with the Universal Eubacteria 16S rRNA gene serving as the reference. Primer sequences for *P. goldsteinii* and universal eubacteria 16S rRNA gene are provided in Table S1 [50, 51].

### Animal experiments

Specific pathogen‐free (SPF) C57BL/6J male mice aged 5−6 weeks were purchased from GemPharmatech and housed in a standard SPF environment. *Tgr5* ^−/−^ mice (on a C57BL/6J background) were generously provided by Professor Bing Du from East China Normal University. Both NCD (Cat# 1010009; Xietong Pharmaceutical) and FFDs utilized in this study were procured from Jiangsu Xietong Pharmaceutical Bio‐engineering. The FFD was modified from the AIN‐93G standard to exclude cellulose (see Table S2 for detailed composition).

Mice were acclimatized and fed for 5 days before the experiments. To establish a pseudo‐sterile mouse model, an antibiotics cocktail (ABX, Ampicillin (Cat# A800429; Macklin), 100 mg/kg; Gentamicin (Cat# G100391; Aladdin), 100 mg/kg; Neomycin (Cat# S17028; Yuanye), 100 mg/kg; Metronidazole (Cat# S17079; Yuanye), 100 mg/kg; Vancomycin (Cat# S17059; Yuanye), 50 mg/kg) was administered daily via gavage for 7 days to deplete natural gut microbiota.

For the first animal experiment involving an IBD model, *P. goldsteinii* was administered to pseudo‐sterile mice at a dose of 2 × 10^8^ CFU/mouse in 200 μL sterile saline once daily for 7 days before initiating 2.5% DSS treatment ad libitum for 7 days, followed by normal drinking water for 4 days. Control groups received equal volumes of sterile saline with or without DSS treatment. Disease activity was monitored daily through body weight changes, stool consistency, rectal bleeding, and calculation of the DAI as described in Table S3. Detailed scoring criteria for histological analysis using hematoxylin and eosin (HE) are provided in Table S4. Similar protocols were followed for subsequent animal experiments, with variations noted below. For the animal experiment 2, mice were administered 1% DSS for 6 days. For animal experiment 4, *P. goldsteinii* administration included co‐treatment with the BSH inhibitor BSH‐IN‐1 (10 mg/kg/d, Cat# CSN67479; CSNpharm), dissolved in a carrier of 5% dimethyl sulfoxide and 95% corn oil. Animal experiment 5 utilized *Tgr5* knockout mice instead of wild‐type C57BL/6J mice, while all other procedures remained consistent with the animal experiment 1.

For the CRC model in the animal experiment 3, microbiota‐depleted mice received *P. goldsteinii* continuously for 7 days before and every 2 days after a single intraperitoneal injection of AOM (10 mg/kg, Cat# A5486; Sigma). Subsequently, mice received 1% DSS in drinking water on Days 5−10, 24−29, and 43−48, with sacrifice occurring on Day 70. Control groups received equivalent volumes of sterile saline with or without AOM/DSS treatment.

In the animal experiment 6 to assess *P. goldsteinii* colonization, pseudo‐sterile mice were randomly assigned to receive either a normal diet or a FFD. One group per diet received *P. goldsteinii* via gavage for 3 days, with fecal samples collected on days 2, 7, 14, 21, 28, 35, and 42. Control groups received saline gavage for 3 days.

At execution, serum was collected after 6 h of fasting. Colon tissues were fixed with polyformaldehyde for various staining techniques (HE, Alcian blue/Periodic‐Acid Schiff (AB/PAS), immunohistochemistry (IHC) for Ki67, ZO‐1, Occludin), or stored at −80°C alongside fecal samples and intestines for further analysis.

### Bacterial culture

*P. goldsteinii* CCUG 48944 was purchased from CCUG (Culture Collection University of Gothenburg), with BNCC (BeNa Culture Collection) serving as the distributor and cultured under strictly anaerobic conditions at 37°C in an anaerobic chamber (Baker Ruskinn). The bacteria were propagated in modified YCFA medium (Cat# HB9212; Hope Bio‐Technology) with 0.5% glucose, 0.001% hemin, 0.001% vitamin K1, 0.05% l‐cysteine and 1% mixed vitamin solution (see Table S5 for details). The colony‐forming units (CFU) of *P. goldsteinii* were estimated on Columbia blood agar plate. Bacteria was collected by centrifugation and diluted to 1 × 10^9^ CFU/mL in sterile saline before gavage.

### Data analysis and graphs

Data were presented as mean ± standard error of the mean (SEM). Statistical analyses and graphs were performed using GraphPad Prism 8 or R‐4.2. Statistical differences between the two values were analyzed using the Mann–Whitney *U* test. Multiple groups comparision were examined for statistical significance using one‐way analysis of variance (ANOVA) or two‐way ANOVA for time‐dependent changes between various groups. Differences in *P. goldsteinii* prevalence between healthy and diseased patients were assessed using the chi‐square test, while Fisher's exact test was used to compare tumor differentiation proportions between groups. Kaplan–Meier curves were compared using the log‐rank test. For analysis of untargeted metabolome data, the permutational multivariate analysis of variance (PERMANOVA) was used to assess differences in Bray–Curtis distance among groups (Anderson, 2001). * or ^#^ *p* < 0.05, ** or ^##^ *p* < 0.01, *** or ^###^ *p* < 0.001, **** or ^####^ *p* < 0.0001, *ns* indicates no significance.

Detailed procedures for sample collection and statistical analysis approaches are available in the Supplementary Information (Tables S6–9).

## AUTHOR CONTRIBUTIONS

**Fujian Qin**: Methodology; investigation; visualization; writing—original draft; data curation. **Mengdi Zhang**: Investigation; validation; writing—review and editing. **Qingling Yang**: Methodology; investigation; writing—review and editing. **Lei Wu**: Resources; investigation. **Tianxiao Mao**: Investigation. **Xingchen Zhou**: Methodology; visualization. **Jing Li**: Writing—review and editing; conceptualization; funding acquisition. **Maode Lai**: Conceptualization; writing—review and editing; funding acquisition.

## CONFLICT OF INTEREST STATEMENT

The authors declare no conflicts of interest.

## ETHICS STATEMENT

All animal procedures were approved (No. 2022−05−044) by the Institutional Animal Care and Use Committee of China Pharmaceutical University.

## Supporting information

The online version contains supplementary figures and tables available.

**Figure S1:** Expression of ZO‐1 and Occludin protein in the colon (Animal Experiment 1).
**Figure S2:** Immunohistochemical determination of ZO‐1 and Occludin protein expression (Animal Experiment 2).
**Figure S3:** High efficiency of *P. goldsteinii* (Pg) colonization under the fiber‐free diet.
**Figure S4:** Total ion chromatograms of mouse fecal samples.
**Figure S5:** Orthogonal projection to latent structure‐discriminant analysis (OPLS‐DA) score plots comparing fecal metabolites.
**Figure S6:** The concentration of bile acid from mice fecal samples under the fiber‐free diet.
**Figure S7:** The concentration of bile acid from mice fecal samples under normal chow diet.
**Figure S8:** mRNA expressions of bile acid receptors in the colon.
**Figure S9:** Genomic DNA from tail snips analyzed by PCR to confirm *Tgr5* knockout.

**Table S1:** Primers used in qPCR analysis and species identification for *P. goldsteinii*.
**Table S2:** The composition of fiber free diet (FFD) used in this study.
**Table S3:** Disease activity index (DAI) scoring rules.
**Table S4:** Hematoxylin‐eosin (HE) staining scoring criteria.
**Table S5:** Mixed vitamin solution.
**Table S6:** Baseline characteristics of public datasets used in this study.
**Table S7:** Baseline characteristics of IBD patients and healthy subjects for *P. goldsteinii* quantification by qPCR from the First Affiliated Hospital of Nanjing Medical University.
**Table S8:** Primer sequences for the measurement of gene expression in mice by real‐time qPCR.
**Table S9:** Detailed ultra‐performance liquid chromatography coupled with a triple quadrupole mass spectrometry (UPLC‐QQQ‐MS/MS) setting for bile acid.

## Data Availability

The data that supports the findings of this study are available in the supplementary material of this article. The public metagenomics data used in this manuscript are available in NCBI of PRJNA398089 (https://www.ncbi.nlm.nih.gov/bioproject/PRJNA398089). The public data that support the findings of this study are openly available in *GMrepo* database (https://gmrepo.humangut.info). The data and scripts used are saved in GitHub https://github.com/Qin-fujian/IMO_2025. Supplementary materials (methods, figures, tables, graphical abstract, slides, videos, Chinese translated version, and update materials) could be found in the online DOI or iMetaOmics http://www.imeta.science/imetaomics/.
