# Supplementary material for: Interactions between Parabacteroides goldsteinii CCUG 48944 and diet ameliorate colitis in mice via regulating gut bile acid metabolism
Source: IMetaOmics. 2025 Mar 18;2(2):e70008. doi: 10.1002/imo2.70008 (PMC12806366; doi:10.1002/imo2.70008)
Supplement: Supplementary file 1 — Figure S1: Expression of ZO‐1 and Occludin protein in the colon (Animal Experiment 1). Figure S2: Immunohistochemical determination of ZO‐1 and Occludin protein expression (Animal Experiment 2). Figure S3: High efficiency of P. goldsteinii (Pg) colonization under the fiber‐free diet. Figure S4: Total ion chromatograms of mouse fecal samples. Figure S5: Orthogonal projection to latent structure‐discriminant analysis (OPLS‐DA) score plots comparing fecal metabolites. Figure S6: The concentration of bile acid from mice fecal samples under the fiber‐free diet. Figure S7: The concentration of bile acid from mice fecal samples under normal chow diet. Figure S8: mRNA expressions of bile acid receptors in the colon. Figure S9: Genomic DNA from tail snips analyzed by PCR to confirm Tgr5 knockout. [file IMO2-2-e70008-s002.docx]

**Supporting Information to:**

**Interactions between *Parabacteroides goldsteinii*** **CCUG 48944 and diet ameliorate colitis in mice** **via regulating gut bile acid metabolism**

**Running title:** *Parabacteroides goldsteinii* CCUG 48944 ameliorate colitis in mice

Fujian Qin^1^, Mengdi Zhang^1^, Qingling Yang^1^, Lei Wu^2^, Tianxiao Mao^3^, Xingchen Zhou^4^, Jing Li^5*^, Maode Lai^1,6 *^

^1^State Key Laboratory of Natural Medicines, School of Basic Medical Sciences and Clinical Pharmacy, China Pharmaceutical University, Nanjing 211198, China

^2^Department of Laboratory Medicine, The First Affiliated Hospital of Nanjing Medical University, Nanjing 211166, China

^3^Department of Pharmacy, Zhongshan Hospital Fudan University, Shanghai 200032, China

^4^Division of (Bio) Pharmaceutics, Institute of Zhejiang University-Quzhou, Quzhou 324000, China

^5^School of Life Science and Technology, China Pharmaceutical University, Nanjing 211198, China

^6^Department of Pathology, Research Unit of Intelligence Classification of Tumor Pathology and Precision Therapy, Chinese Academy of Medical Science, Key Laboratory of Disease Proteomics of Zhejiang University School of Medicine, Hangzhou 310058, China

*Correspondence: [lmd@cpu.edu.cn](mailto:lmd@cpu.edu.cn) (Maode Lai), [lj_cpu@126.com](mailto:lj_cpu@126.com) (Jing Li)

**SUPPLEMENTARY METHODS**

**Public metagenomic sequence datasets**

Metagenomic sequence data from American individuals was made publicly available (PRJNA398089, Table S6). The metagenomic analysis method is consistent with before. Initially, the raw sequencing reads were carefully processed and refined using the FASTX Toolkit (v 0.0.13). The high-quality microbiome sequencing data were then assembled using SOAPdenovo2 (v 2.04). After this assembly phase, contigs with a minimum length of 500 base pairs were selected for gene prediction, which was performed using MetaGeneMark (v 2.8).

*Parabacteroides goldsteinii* (*P. goldsteinii*) prevalence data were obtained from the *GMrepo* public database (<https://gmrepo.humangut.info>). The keyword " *Parabacteroides goldsteinii*" was used to search the database, and data were filtered to include only healthy individuals and those with colitis

**Fecal samples collection**

Inflammatory bowel disease (IBD) was diagnosed following the consensus guidelines on the diagnosis and treatment of IBD (2018, Beijing). Criteria included persistent or recurrent diarrhea, mucopurulent bloody stool with abdominal pain, colonoscopy results, and histopathological evidence of inflammatory cell infiltration, crypt structural changes, mucosal erosion, and ulcer formation in mucosal biopsies or surgically excised specimens.

Exclusion criteria included recent antibiotic or probiotic treatment within the past month, acute infectious enteritis, amebic enteropathy, intestinal schistosomiasis, drug-associated enterocolitis, or ulcerative colitis (UC) combined with Clostridium difficile or CMV infection.

The study recruited 14 individuals with UC, 22 individuals with Crohn's disease (CD), and 13 matched healthy controls from the First Affiliated Hospital of Nanjing Medical University (Nanjing, China). Gender, age, and clinical characteristics are summarized in Table S7. After fasting for at least 8 hours, fecal samples were collected immediately and stored at -80°C. The study received approval from the Ethics Committee of the First Affiliated Hospital of Nanjing Medical University.

**Real‑time quantitative PCR**

Total RNA was extracted from the colon tissues of mice by using RNA-easy isolation reagent (Cat# R701, Vazyme, Nanjing, China). Concentration was measured using the Nano-100. Then 1 μg of total RNA was reversely transcribed using HiScript II Q RT SuperMix for qPCR (+gDNA wiper) (Cat# R223, Vazyme, Nanjing, China). The relative mRNA expression was normalized relative to glyceraldehyde-3-phosphate dehydrogenase (*Gapdh*) using the 2^−ΔΔCt^ method. All oligonucleotide primers target genes and internal reference genes are listed in Table S8 [1–6].

**Tissue histology and immunohistochemical analyses**

For histological studies, the distal colon tissues were fixed in 4% neutral buffered formalin overnight. Then send it to corporate (Servicebio, China) for embedding and tissue staining. In particular, HE staining is used to evaluate histological scores by a qualified and blinded pathologist, AB-PAS staining shows mucopolysaccharides and goblet cells, ki67 staining detects cell proliferation, and ZO-1 & Occludin were measured to further investigate intestinal barrier integrity. Images were captured using NanoZoomer 2.0. A fluorescence in situ hybridization (FISH) experiment is used to detect the colonization of *P. goldsteinii* in the distal colon, and specific fluorescent probes are listed in Table S1. NCBI blast results show that the probe has reliable specificity (Figure S3D).

**Untargeted assay**

Fecal metabolites were analyzed using ultra-performance liquid chromatography-quadrupole time-of-flight mass spectrometry (UPLC-Q/TOF-MS, Agilent 6545) with modifications based on previous methods [7].

For the extraction of fecal metabolites, the stool samples were weighed and dissolved in 50% acetonitrile solution at a volume ratio of 1:20 (mg/μL). The samples were ground, centrifuged at 13,000 g for 10 minutes at 4°C, and the supernatant was collected. An equal volume of acetonitrile containing the internal standard (100 ng/mL L-2-chlorophenylalanine) was added, and the mixture was centrifuged twice for 15 minutes each. The final supernatant was prepared for measurement.

Chromatographic separation was achieved using a Waters HSS T3 column (100 × 2.1 mm, 1.8 μm particle size). Data acquisition was performed with Agilent 6545 MassHunter Workstation software (version B.08.00). For positive ion mode (ESI+), the mobile phases were 0.1% formic acid in acetonitrile (B) and water (A). For negative ion mode (ESI-), the mobile phases were acetonitrile and water, both containing 5 mmol/L ammonium acetate. The gradient elution program was as follows: 0–2 minutes, 5% solvent B; 2–3 minutes, 5%–30% solvent B; 3–10 minutes, 30%–90% solvent B; 10–11 minutes, 90%–100% solvent B; 11–13 minutes, 100% solvent B. The total run time was 13 minutes, with an additional 4 minutes for post-processing. The flow rate was 0.3 mL/min, the injection volume was 2 μL, and the column temperature was 45°C.

The fragmentor voltage was set at 120 V, skimmer voltage at 65 V, drying gas flow rate at 12 L/min, temperature at 350°C, sheath gas flow rate at 12 L/min, and temperature at 375°C. Reference masses at m/z 121.0508 and 922.0098 (ESI+), and 112.9855 and 980.0163 (ESI-) were used for accurate mass calibration.

Quality control (QC) samples, comprising a mix of all samples, were used to calibrate the analytical blocks and assess technical accuracy. To ensure a stable baseline, 5 blank samples were injected before analyzing the sample sequence. QC and blank samples were run after every 10 injections of prepared samples. Data were collected using the MassHunter workstation software, exported to m/z 100–1000, and preprocessed using the R package XCMS. Ion features present in less than 80% of the samples were excluded. Principal component analysis (PCA) was used to visualize the metabolic profiles of each group. The princomp function from the stats package was used for dimensionality reduction, extracting the first two principal components for subsequent analysis. Potential differential ions were identified based on the orthogonal partial least squares discriminant analysis (OPLS-DA) model using criteria of variable importance in projection (VIP) value > 1.0, absolute value of log_2_ fold-change > 1, and false discovery rate (FDR) < 0.05. OPLS-DA was performed using the ropls package in R. Data preprocessing involved centering and scaling using the scale function. The opls function was used to build the model. Heatmaps were generated using the pheatmap package in R. Differential metabolites were tentatively identified using the Human Metabolome Database (HMDB) and used for pathway analysis via MetaboAnalyst 5.0 (<https://www.metaboanalyst.ca/>).

**Quantification of biles acids**

The process for extracting fecal bile acids was similar to that used for UPLC-Q/TOF-MS, with the internal standard replaced by the corresponding bile acid isotope. Analysis of bile acids was conducted using ultra-performance liquid chromatography coupled with a triple quadrupole mass spectrometry (UPLC-QQQ-MS/MS, Agilent 6495) with modifications from previous methods [8]. Briefly, a 5 μL extract was injected into a Waters HSS T3 column operating in negative ion electrospray mode. Liquid chromatography separation was performed at a flow rate of 0.4 mL/min using mobile phase A (0.1% formic acid in water) and mobile phase B (0.1% formic acid in acetonitrile). The gradient conditions were: 0−1 min, 5%−25% B; 1−9 min, 25%−30% B; 9−10 min, 30%−40% B; 10−17 min, 40%−45% B; 17−18.5 min, 45%−95% B; and 18.5−20.5 min, 95% B. Data were collected using targeted multiple reaction monitoring (MRM) methods.

A 5−7 point calibration curve was constructed for each bile acid to calculate absolute concentrations, with detailed information provided in Table S9. Both untargeted and targeted metabolomics analyses of mouse stool were conducted at the Center for Target Discovery, the public experimental platform of China Pharmaceutical University.

**REFERENCES**

1. Fu, Ting, Sally Coulter, Eiji Yoshihara, Tae Gyu Oh, Sungsoon Fang, Fritz Cayabyab, et al. 2019. “FXR regulates intestinal cancer stem cell proliferation.” *Cell* 176: 1098–1112.e1018. <https://doi.org/10.1016/j.cell.2019.01.036>

2. Castellanos-Jankiewicz, Ashley, Omar Guzmán-Quevedo, Valérie S Fénelon, Philippe Zizzari, Carmelo Quarta, Luigi Bellocchio, et al. 2021. “Hypothalamic bile acid-TGR5 signaling protects from obesity.” *Cell Metabolism* 33: 1483–1492.e1410. <https://doi.org/10.1016/j.cmet.2021.04.009>

3. Yan, Tingting, Yuhong Luo, Yangliu Xia, Keisuke Hamada, Qiong Wang, Nana Yan, et al. 2021. “St. John's Wort alleviates dextran sodium sulfate-induced colitis through pregnane X receptor-dependent NFkappaB antagonism.” *FASEB Journal* 35: e21968. <https://doi.org/10.1096/fj.202001098R>

4. Nakamichi, Yuko, Nobuyuki Udagawa, Kanji Horibe, Toshihide Mizoguchi, Yoko Yamamoto, Takashi Nakamura, et al. 2017. “VDR in osteoblast-lineage cells primarily mediates vitamin D treatment-induced increase in bone mass by suppressing bone resorption.” *Journal of Bone and Mineral Research* 32: 1297–1308. <https://doi.org/10.1002/jbmr.3096>

5. Choi, You-Jin, Dong Zhou, Anne Caroline S Barbosa, Yongdong Niu, Xiudong Guan, Meishu Xu, et al. 2018. “Activation of constitutive androstane receptor ameliorates renal ischemia-reperfusion-induced kidney and liver Injury.” *Molecular Pharmacology* 93: 239–250. <https://doi.org/10.1124/mol.117.111146>

6. Zhang, Jianan, Morgan E Walker, Katherine Z Sanidad, Hongna Zhang, Yanshan Liang, Ermin Zhao, et al. 2022. “Microbial enzymes induce colitis by reactivating triclosan in the mouse gastrointestinal tract.” *Nature Communications* 13: 136. <https://doi.org/10.1038/s41467-021-27762-y>

7. Li, Jiankang, Jing Li, Huan Wang, Lian-Wen Qi, Yimin Zhu, Maode Lai. 2019. “Tyrosine and glutamine-leucine are metabolic markers of early-stage colorectal cancers.” *Gastroenterology* 157: 257–259. <https://doi.org/10.1053/j.gastro.2019.03.020>

8. Song, Ziwei, Shuo Feng, Xingchen Zhou, Zhengxing Song, Jing Li, Ping Li. 2023. “Taxonomic identification of bile salt hydrolase‐encoding lactobacilli: Modulation of the enterohepatic bile acid profile.” *iMeta* 2: e128. <https://doi.org/10.1002/imt2.128>

**Supplementary figure**

**
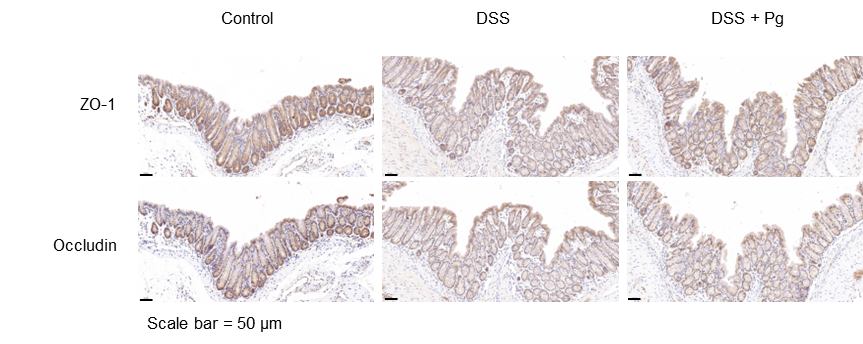
**

**Figure S1 Expression of ZO-1 and Occludin protein in the colon (Animal Experiment 1).** Representative images from immunohistochemistry assays in *n* = 3 biologically independent animals fed a normal chow diet (NCD, Animal Experiment 1, scale bars: 50 μm). Pg: *P. goldsteinii/Parabacteroides goldsteinii.*

**
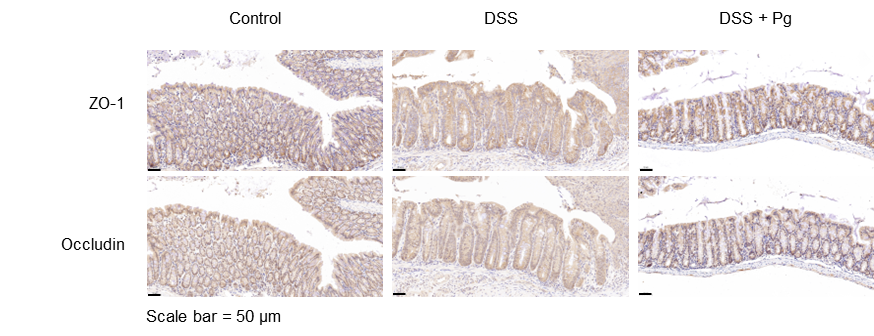
**

**Figure S2** **Immunohistochemical determination of ZO-1 and Occludin protein expression (Animal Experiment 2).** Representative images from *n* = 3 biologically independent animals fed a fiber-free diet (FFD, Animal Experiment 2, scale bars: 50 μm).


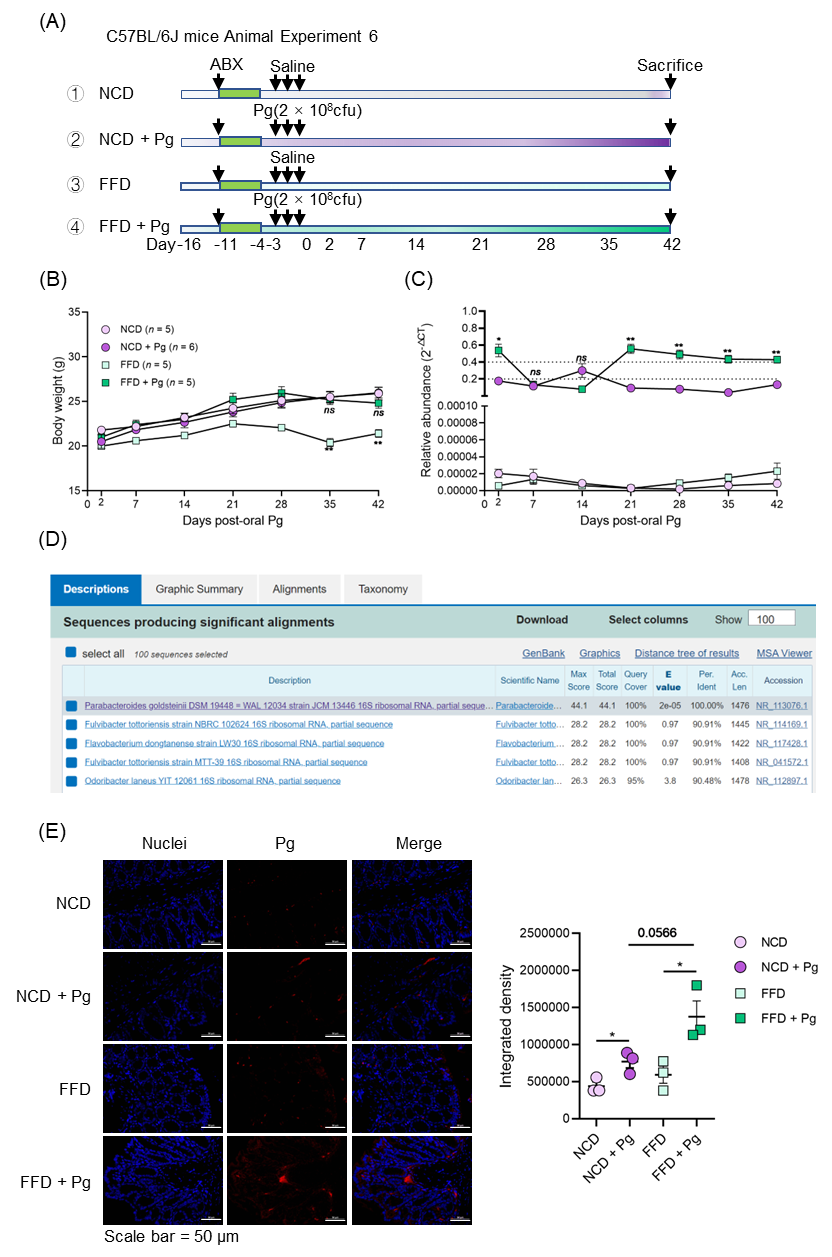


**Figure S3 High efficiency of *P. goldsteinii* colonization under the fiber-free diet.** (A) Experimental scheme for ABX treatment and *P. goldsteinii* administration. (B) Body weight changes after *P. goldsteinii* gavage. Two-way ANOVA with Tukey’s multiple comparision test. ***p* < 0.01 (NCD group vs. FFD group); *ns* indicates no significance (NCD + Pg group vs. FFD + Pg group). (C) Fecal *P. goldsteinii* levels at various time points. Two-way ANOVA with Tukey’s multiple comparision test. **p* < 0.05, ***p* < 0.01, *ns* indicates no significance (NCD + Pg group vs. FFD + Pg group) (D) Specificity of the fluorescence in situ hybridization (FISH) probe for *P. goldsteinii* detection evaluated by BLAST through the NCBI database. (E) Colonic colonization of *P. goldsteinii* determined by FISH assay (scale bar, 50 μm). Red staining (5’Cy3-labeled) indicates *P. goldsteinii*; blue DAPI staining shows cell nuclei. *n* = 3 mice/group. One-way ANOVA with Tukey’s multiple comparision test. **p* < 0.05.


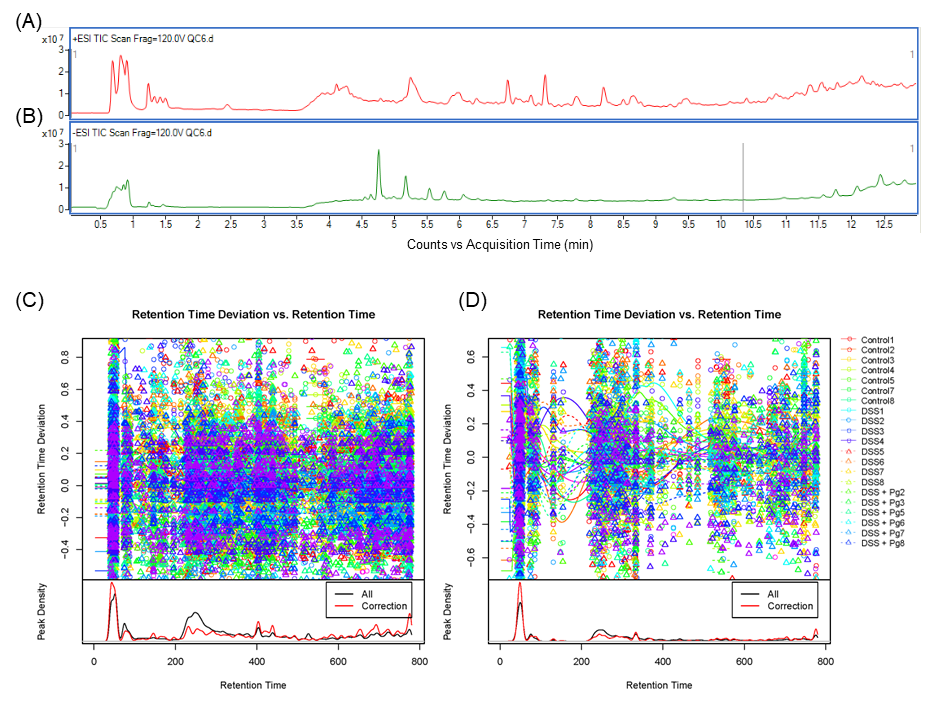


**Figure** **S4** **Total ion chromatograms of mouse fecal samples.** (A and B) Positive ion mode (A) and negative ion mode chromatograms (B). (C and D) Variation in retention time in positive ion mode (C) and negative ion mode (D) (Animal Experiment 2).


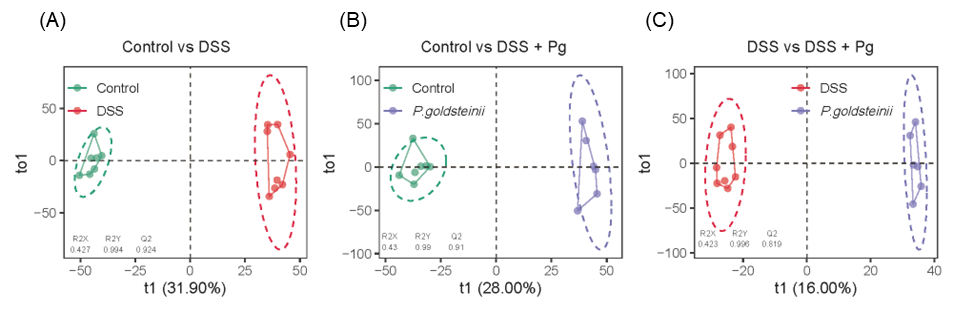


**Figure S5 Orthogonal projection to latent structure-discriminant analysis (OPLS-DA) score plots comparing fecal metabolites**. (A) Control vs. DSS groups. (B) Control vs. DSS + Pg groups. (C) DSS vs. DSS + Pg groups (Animal Experiment 2).


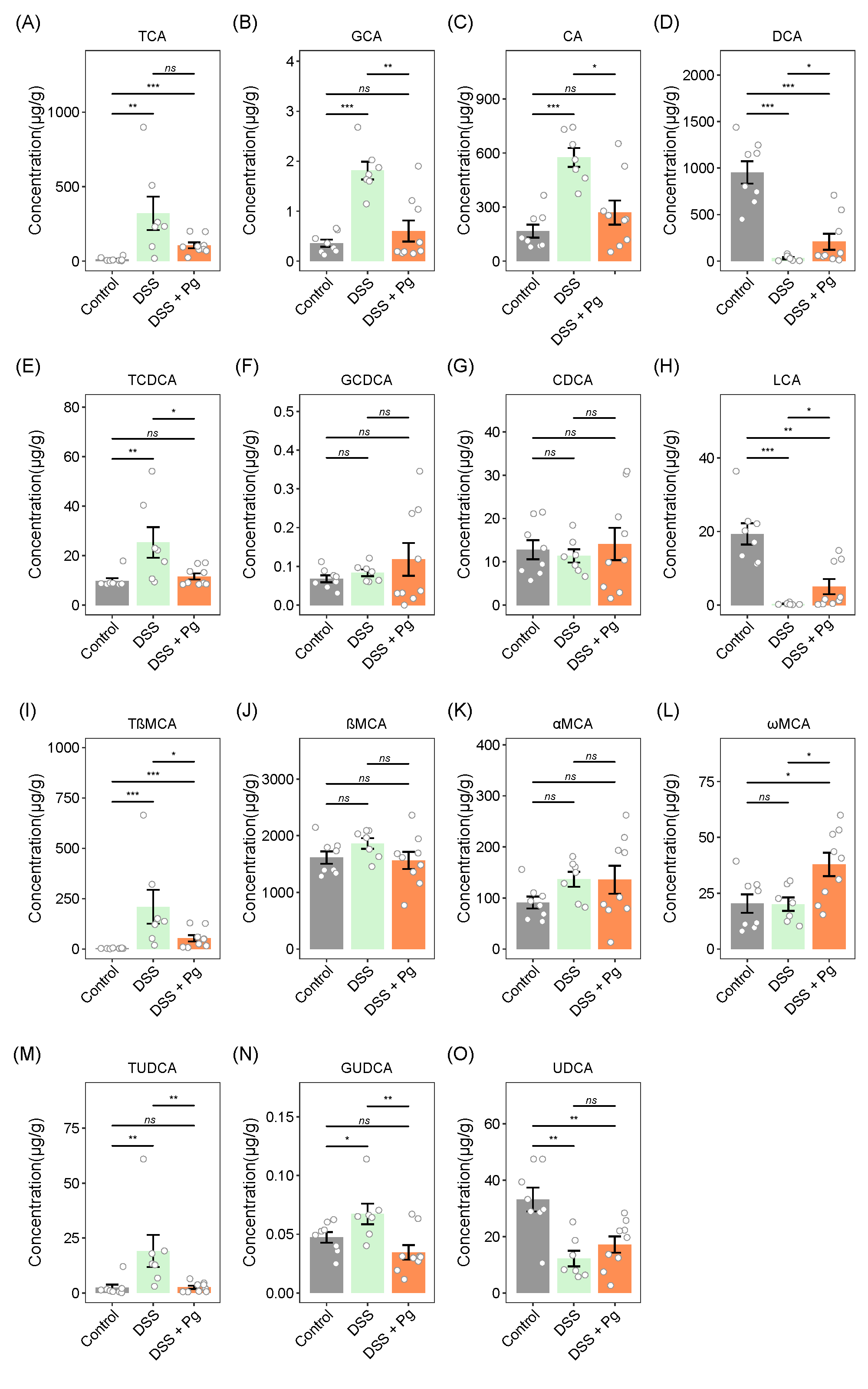


**Figure S6 The concentration of bile acid from mice fecal samples under the fiber-free diet.** (A) Taurocholic acid (TCA) content. (B) Glycocholic acid (GCA) content. (C) Cholic acid (CA) content. (D) Deoxycholic acid (DCA) content. (E) Taurochenodeoxycholic acid (TCDCA) content. (F) Glycochenodeoxycholic acid (GCDCA) content. (G) Chenodeoxycholic acid (CDCA) content. (H) Lithocholic acid (LCA) content. (I) Tauro β muricholic acid (TβMCA) content. (J) β Muricholic acid (βMCA) content. (K) α Muricholic acid (αMCA) content. (L) ω Muricholic acid (ωMCA) content. (M) Tauroursodeoxycholic acid (TUDCA) content. (N) Glycoursodeoxycholic acid (GUDCA) content. (O) Ursodeoxycholic acid (UDCA) content. One-way ANOVA with Tukey’s multiple comparision test. *, **, and *** denote *p* < 0.05, 0.01, and 0.001, respectively; *ns* denotes no significant difference (Animal Experiment 2).


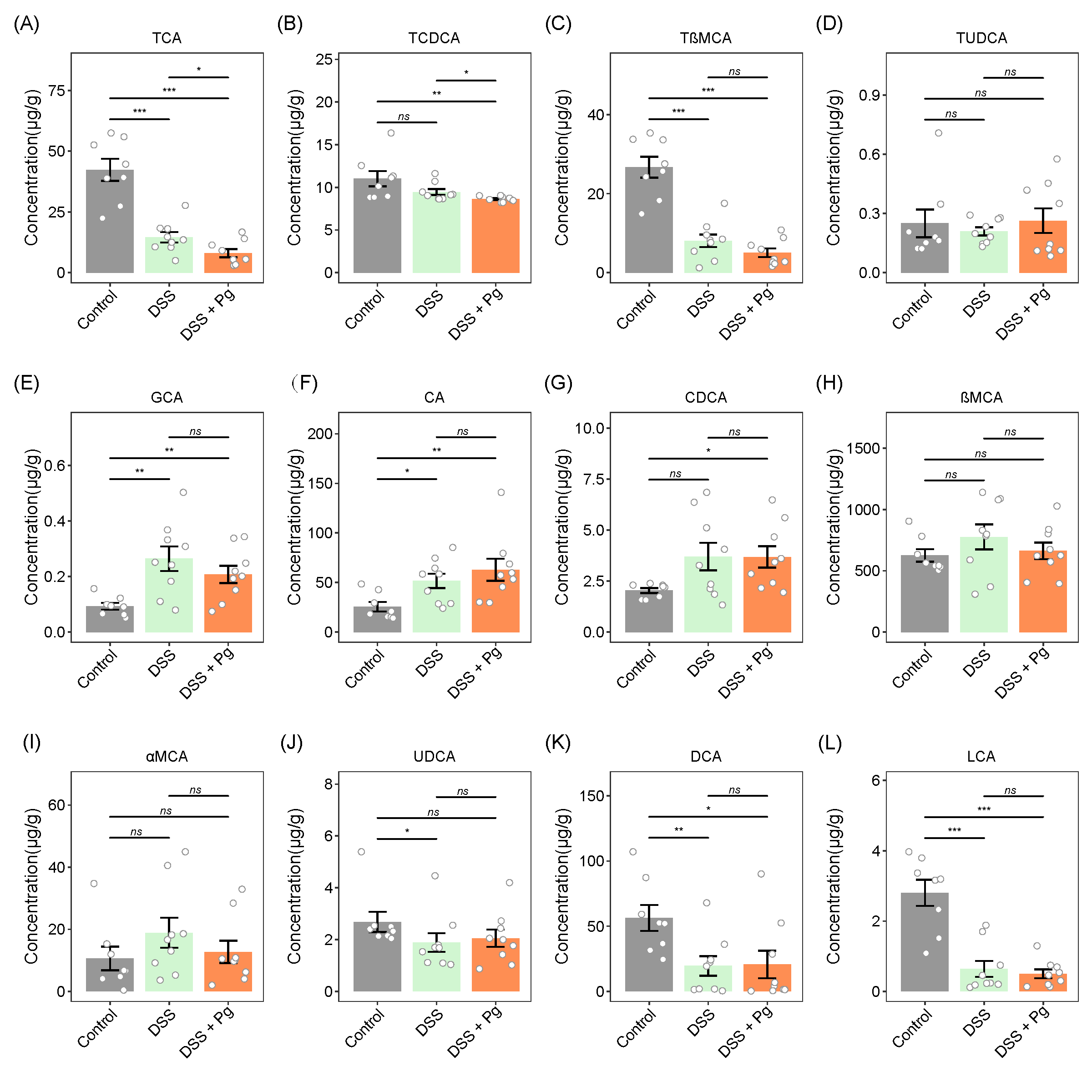


**Figure S7 The concentration of bile acid from mice fecal samples under normal chow diet.** (A) TCA content. (B) TCDCA content. (C) TβMCA content. (D) TUDCA content. (E) GCA content. (F) CA content. (G) CDCA content. (H) βMCA content. (I) αMCA content. (J) UDCA content. (K) DCA content. (L) LCA content. One-way ANOVA with Tukey’s multiple comparision test. *, **, and *** denote *p* < 0.05, 0.01, and 0.001, respectively; *ns* denotes no significant difference (Animal Experiment 1).


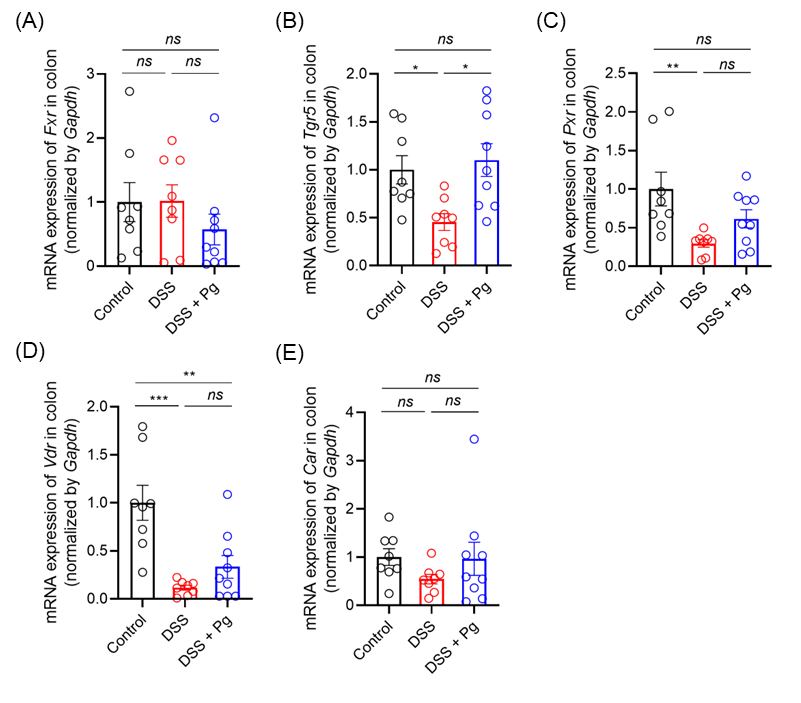


**Figure S8** **mRNA expressions of bile acid receptors** **in the colon.** (A) Farnesoid X receptor (*Fxr*) level. (B) Takeda G-protein-coupled receptor 5 (*Tgr5*) level. (C) Pregnane X receptor (*Pxr*) level. (D) Vitamin D receptor (*Vdr*) level. (E) Constitutive androstane receptor (*Car*) level. One-way ANOVA with Tukey’s multiple comparision test. *, **, and *** denote *p* < 0.05, 0.01, and 0.001, respectively; *ns* denotes no significant difference (Animal Experiment 2).


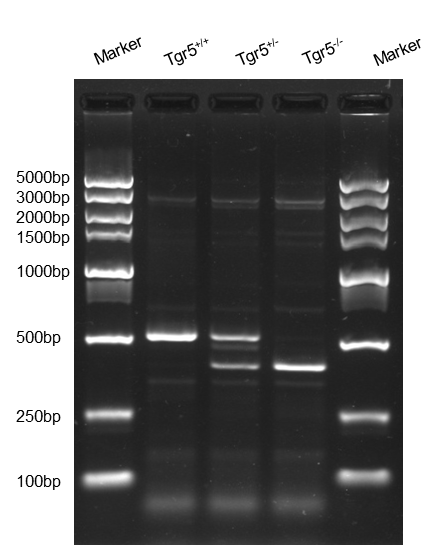


**Figure S9 Genomic DNA from tail snips analyzed by PCR to confirm *Tgr5* knockout.**
